# Fungus Hematodes

**Published:** 1850-09

**Authors:** 


FUNGUS HEMATODES.

In the following letter, Dr. Debow, of Hartsville, Tennessee, refers to an operation for this intractable affection, which seems to have been crowned with success. Dr. Debow promises to communicate further details of the interesting case:

HARTSVILLE, August 27, 1850.

PROFESSOR YANDELL—DEAR SIR: I had intended before this, to have written to you, giving an account of a case of fungus hematodes, which involved some three inches of the ulna of the right arm, so much so as to render it necessary after taking away the tumor, to take out at least three or three and a half inches of the bone before it could be caused to heal. A second operation with the knife was necessary, some two months after the first, after which, as the fungus would sprout up, it was met and finally conquered by the local application of finely pulverized corrosive sublimate, so soon as all evidences of returning fungus was manifest, the annexed portion of the bone having all been taken away. No other application was made but Peruvian bark rubbed up in Basilicon ointment, until a cure was effected. I should have remarked that so much of the corrosive sublimate was absorbed as to cause the patient to spit very freely for some two or three weeks. May not this effect have had much to do in the cure? He is now in fine health—his arm perfectly well, the part having partially filled with a sound osseous deposit, which renders it almost as strong as in his former life.

I say I had intended to give you this case before now, but feared to do so earlier lest the disease might return—some eighteen months have now elapsed since it seemed to heal, and at this time has every appearance of remaining well.

